# Is the use of diagnostic imaging and the self-reported clinical management of low back pain patients influenced by the attitudes and beliefs of chiropractors? A survey of chiropractors in the Netherlands and Belgium

**DOI:** 10.1186/s12998-023-00523-y

**Published:** 2024-01-08

**Authors:** Brenda van der Vossen, Annemarie de Zoete, Sidney Rubinstein, Raymond Ostelo, Michiel de Boer

**Affiliations:** 1 Department of Health Sciences, Faculty of Science, VU University Amsterdam, Amsterdam Movement Sciences Research Institute Amsterdam, Amsterdam, The Netherlands; 2 Department of Epidemiology and Data Science, Amsterdam University Medical Centre, Location Vrije Universiteit Amsterdam, Amsterdam, The Netherlands; 3 Department of Primary- and Long-Term Care, UMCG, Antonius Deusinglaan 1, 9713 AV Groningen, The Netherlands

**Keywords:** Low back pain, Chiropractic, Guideline adherence, Attitudes and beliefs, Management, Latent profile analysis

## Abstract

**Background:**

No previous studies have examined the association between attitudes and beliefs of chiropractors and their adherence to low back pain (LBP) guidelines. The aim of this study is: (1) to assess the attitudes and beliefs towards the management of LBP of Dutch and Belgian chiropractors; and (2) to investigate the association of these attitudes and beliefs on the use of diagnostic imaging and on the adherence to diagnostic guidelines and guidelines in the management of patients with LBP.

**Methods:**

**Study design:**

Cross-sectional study using a web-based questionnaire in chiropractic private practices in the Netherlands and Belgium. The survey included sociodemographic characteristics, use of diagnostic imaging, the Pain Attitude and Beliefs Scale-Physiotherapists (PABS.PT) and 6 vignettes (3 acute and 3 chronic LBP patients). We used Latent Profile Analysis (LPA) to categorise the chiropractors into clusters depending on their PABS.PT outcome, whereby the classes differed primarily on the biomedical score. We used linear, logistic, and mixed models to examine the associations between these clusters, and adherence to the recommendations of guidelines on: (1) diagnostic imaging use, and (2) management of LBP (i.e. advice on activity, treatment, return-to-work, and bedrest).

**Results:**

The response rate of the Dutch and Belgian chiropractors was 61% (n = 149/245) and 57% (n = 54/95), respectively. The majority of chiropractors scored midrange of the biomedical scale of the PABS.PT. Three clusters were identified using LPA: (1) high biomedical class (n = 18), (2) mid biomedical class (n = 117) and (3) low biomedical class (n = 23).

Results from the vignettes suggest that chiropractors in the high biomedical class better adhere to diagnostic imaging guidelines and to LBP guidelines when it concerns advice on return-to-work and activity compared to the other two classes. However, no differences were identified between the classes for treatment of LBP. All chiropractors adhered to the guidelines’ recommendation on bedrest.

**Conclusion:**

The high biomedical class demonstrated better overall adherence to the practice guidelines for the management of LBP and diagnostic imaging than the other classes. Due to the small numbers for the high and low biomedical classes, these results should be interpreted with caution.

**Supplementary Information:**

The online version contains supplementary material available at 10.1186/s12998-023-00523-y.

## Introduction

Low back pain (LBP) is a common and costly burden worldwide [1]. For the treatment and management of LBP, there is a diversity of treatment options, which have been outlined in numerous international guidelines [2, 3]. These guidelines have been developed on national and international levels to improve effectiveness of health care, to assist in triaging for red flags, and to reduce the risk of unintended adverse effects or chronic disability [2, 5–8]. One treatment option recommended in LBP guidelines is spinal manipulative therapy (SMT), which is, among others, provided by chiropractors [9]. Similarly, guidelines for diagnostic imaging have also been developed, amongst others to minimise unnecessary radiation exposure [4, 10, 11]. Diagnostic imaging is an integral part of chiropractic practice, and imaging of the lumbosacral spine is the most frequently used [12]. Approximately half of the patients presenting to a chiropractor complain of LBP; therefore, it would appear to be a condition that chiropractors commonly treat [13].

Guideline recommendations are infrequently followed by practitioners as several international studies have demonstrated [14, 15]. Attitudes and beliefs of practitioners have been named as one of the factors contributing to non-adherence to these guidelines [14], which includes the practitioner’s orientation towards a biomedical or a biopsychosocial treatment model. In a biomedical orientation, the practitioner believes that pain derives from tissue damage, whereas in the biopsychosocial orientation, social and psychological factors are thought to influence pain as well [16].

Studies which examined general practitioners and physiotherapists' attitudes and beliefs in relation to their adherence to the recommendations in practice suggested that practitioners with a higher biomedical orientation were less likely to adhere to the recommendations than practitioners with a higher biopsychosocial orientation (or lower biomedical orientation) [14, 15]. In the only study that we are aware of that assessed the orientation of chiropractors, however, the authors did not investigate the relation between this orientation and adherence to guidelines [17]. In short, no previous studies have examined the association between attitudes and beliefs of chiropractors and their adherence to guidelines. Therefore, the aim of the current study is twofold. First, we will assess the attitudes and beliefs towards the management of low back pain (LBP) of Dutch and Belgian chiropractors; and secondly, we will investigate the association between chiropractors’ attitudes and beliefs and a) the adherence to the recommendation of clinical guidelines on LBP and to diagnostic imaging guidelines and b) self-reported frequency of using diagnostic imaging.

## Methods

This article has been written along the CROSS reporting guidelines (Additional file 1: Cross reporting guideline).

## Design and setting

The study was designed as a cross-sectional observational study, using a web-based questionnaire (SurveyMonkey).

In 2013, all chiropractors in the Netherlands (n = 245), who were registered with the Chiropractic Registration Board for the Netherlands (‘Stichting Chiropractie Nederland’(SCN)), and all Belgian chiropractors (n = 95) registered with the Belgian Chiropractic Union (BCU; ‘Belgische Vereniging van Chiropractors’), were invited to participate via a web-based questionnaire. After 3 weeks, an email reminder was sent and after 6 weeks a reminder telephone call was made to those chiropractors who had not yet completed the questionnaire. Each chiropractor received one link, that could not be re-used after finishing and sending the (first) questionnaire. The questionnaire contained questions on demographics, the validated Pain Attitude and Beliefs Scale-Physiotherapists (PABS.PT) and six patient vignettes. Responses were coded and not traceable to the responding chiropractor.

## Questionnaire

### Prior to data collection

Prior to data collection, the questionnaire was pre-tested in a pilot study using three Dutch chiropractors. This led to minor textual changes in the questions. Approximately 40 min were required to complete the questionnaire (Additional file 2: Survey). The questionnaire was designed in a manner that participants could only go to the next question if they had completed the previous one.

### Sociodemographics and practice information

The questionnaire started with questions about demographics (eg. age, gender) and general characteristics (eg. years in practice, postgraduate training, type of practice) (Additional file 2: Survey).

### Attitudes and beliefs

The PABS.PT was developed and validated as a tool to assess the biomedical and biopsychosocial treatment orientation with regards to (non-specific) LBP [16, 18]. The PABS.PT was included as a measure of the chiropractors’ attitudes to LBP. It consists of 20 items, each rated on a six point Likert scale (‘totally disagree’ to ‘totally agree’) with fourteen items on the biomedical subscale (score range 14–84) and six on the bio-psychosocial subscale (score range 6–36). A higher score on the subscales indicates a stronger biomedical or bio-psychosocial therapy orientation respectively. Scores for the PABS.PT were calculated according to the methods described by its developers, i.e. simple summation of the items in each subscale [16].

### Guideline adherence

We used six patient vignettes (Additional file 2: Survey) reflecting three patients with acute LBP and three with chronic LBP whom chiropractors would typically see in their practices. The vignettes were taken from previous studies and adapted to the Dutch and Belgian situation [19]. For each clinical vignette, the chiropractors were asked if they would order diagnostic imaging and how they would manage the patient.

With regards to treatment, the options in the vignettes included no intervention, chiropractic manipulative therapy, exercise, education, spinal traction, psychosocial evaluation and non-exercise modalities. In addition, the vignettes included questions on advice to stay active, avoid bed rest, and return to work, as this information has been recognized in previous studies as potential barriers to practitioners making decisions consistent with guideline recommendations [4, 8].

These responses were classified by the authors as being ‘strictly in line with guideline recommendations’, ‘broadly in line with guideline recommendations’ or ‘not in line with guideline recommendations’ (Table 1). The appropriateness of responses was defined a priori by the project group using recommendations of the international chiropractic and multidisciplinary guidelines [20, 21] [22, 23]. The Dutch multidisciplinary guideline for the management of LBP [22] was used to rule out conflicting evidence or when the recommendation was not clear. Five chiropractors from the United States, Belgium and Australia working in clinical practice, with multiple years of experience in chiropractic research and not participating in the survey, were asked to review our classification of the responses. After minor revisions, they agreed with the classification. For the analysis of the data on adherence to the guidelines in the vignettes, ‘strictly in line with the guidelines’ and ‘broadly in line with the guidelines’ were combined to the category ‘in line with the guidelines’.

**Table 1:** Classification of the responses to the six vignettes

Question	Vignette	Response options on questionnaire	Authors’ classification of responses
Treatment(s) offered at this visit	Vignette 1 and 3	‘no intervention’ or ‘chiropractic adjustment’	Strictly in line with the guideline recommendations
		‘no intervention’ or ‘chiropractic adjustment + one other treatment option	Broadly in line with the guideline recommendations
		2 treatment options other than ‘no intervention’ or ‘chiropractic adjustment’	Not in line with the guideline recommendations
	Vignette 2	‘no intervention’ or ‘chiropractic adjustment’ and/or ‘exercise’	Strictly in line with the guideline recommendations
		‘no intervention’ or ‘chiropractic adjustment’ and/or ‘exercise’ + one other treatment option	Broadly in line with the guideline recommendations
		2 treatment options other than ‘no intervention’ or ‘chiropractic adjustment’ and/or ‘exercise’	Not in line with the guideline recommendations
	Vignette 4, 5, 6	‘no intervention’ or ‘chiropractic adjustment’, ‘exercise’ and/or ‘psychological evaluation’	Strictly in line with the guideline recommendations
		‘no intervention’ or ‘chiropractic adjustment’, ‘exercise’ and/or ‘psychological evaluation’ + one other treatment option	Broadly in line with the guideline recommendations
		2 treatment options other than ‘no intervention’ or ‘chiropractic adjustment’, ‘exercise’ and/or ‘psychological evaluation’	Not in line with the guideline recommendations
Advice to return to work	All vignettes	Return to work	Strictly in line with the guideline recommendations
		Return to part-time or light duties	Broadly in line with the guideline recommendations
		Be off work for a further … weeks (stating number of weeks)	
		Be off work until pain has improved	Not in line with the guideline recommendations
		Be off work until pain has completely resolved	
Advice to stay active	All vignettes	Perform usual activities	Strictly in line with the guideline recommendations
		Perform activities within the patient’s tolerance	Broadly in line with the guideline recommendations
		Perform only pain free activities	Not in line with the guideline recommendations
		Limit all physical activities until the pain disappears	
Investigations ordered at this visit	Vignette 1 and 3	‘no referral for diagnostic imaging’	Strictly in line with the guideline recommendations
		Other options except ‘no referral for diagnostic imaging’	Not in line with the guideline recommendations
	Vignette 2, 4, 5, 6	‘no referral for diagnostic imaging’	Strictly in line with the guideline recommendations
		Referring for radiographs	Broadly in line with the guideline recommendations
		Referring for MRI scan	Not in line with the guideline recommendations

### Use of diagnostic imaging

In this section, questions were asked on the percentage of requests by chiropractors for diagnostic imaging of patients in their practice(s).

### Knowledge of Practice guidelines

Information on the self-reported knowledge of LBP guidelines by chiropractors was collected by yes/no response options.

## Analysis of data

### Attitudes and beliefs

Chiropractors’ characteristics and the biomedical and biopsychosocial subscales of the PABS.PT score were described using means and standard deviations (SDs) for continuous data and percentages for categorical data.

Latent Profile analysis (LPA) of the biomedical and biopsychosocial subscales of the PABS.PT score was used to define different profiles of attitudes and beliefs among chiropractors [24]. We decided a priori that two or three classes would provide the most useful outcomes, as the number of participants was insufficient for more classes. We identified two outliers. Analyses without these outliers led to considerably better interpretable classifications. Therefore, we decided to exclude these two outliers during the formation of the different latent profile models. Different models, without these two outliers, were examined, varying from fully restricted to models that allowed varying variance parameter estimates between classes and unstructured covariance structures. The choice for a final model was based on the combination of best fit (AIC and BIC), the entropy of the model, and the face validity of the classes (Additional file 3: LPA steps of analysis) [24]. The final outcome was tested for robustness by running several models with different numbers of random starts. This showed that our results were robust. The two outliers were then manually added to one of the 3 classes that we identified by our model, based on their biomedical and biopsychosocial scores (Additional file 4: manual addition of outliers).

### Adherence to guidelines in relation to attitudes and beliefs

We ran several logistic mixed models in which we analysed the association between the three LPA classes and the adherence to the LBP guidelines with respect to: (1) treatment, (2) return-to-work, (3) activity and (4) bedrest and (5) guidelines on diagnostic imaging. We performed separate analysis for a) all six vignettes, b) the three vignettes on acute low back pain, and c) the three vignettes on chronic low back pain. As all chiropractors gave advice on bedrest in line with the guidelines, this question was not further analysed.

In the logistic mixed models, we included a random intercept on the chiropractor level in the model. This method was used to allow for the correlation of responses within each individual chiropractor, as each chiropractor answered six vignettes and these responses cannot be seen as six independent responses. Fixed effects for LPA classes were added to the model. These fixed effects estimated the univariable associations between these LPA classes and adherence to guidelines by chiropractors (dependent variables). We present odds ratios (OR’s) and 95% Confidence Intervals (CI’s), complemented by prevalences expressed in percentages and their 95% CI’s. Prevalences were calculated by using the following formula: p = e^β/(1 + e^β)*100% for the reference category. The prevalences for the other classes were calculated by changing the reference category in the analysis. In this study these percentages describe the estimated percentages of subgroups of chiropractors (e.g. biomedical orientated) adhering to the imaging guidelines. For ORs, predefined thresholds for weak (OR < 1.6), medium (1.6 < OR < 3.5) and strong (OR > 3.5) associations were used [25]. Univariable linear regression analysis was used for associations between the self-reported amount of requesting diagnostic imaging and the three LPA classes. A univariable logistic regression model was used to examine the association between the LPA classes and self-reported familiarity with (LBP) practice guidelines.

All analyses were performed in Statistical Package for Social Sciences for Windows (SPSS version 25) except for the LPA, which was performed in Stata 16.1.

## Results

Of the questionnaires sent out (n = 340), 203 questionnaires (60%) were returned. Only participants who filled out the entire questionnaire, including all questions on the PABS.PT questionnaire (n = 158) were included, therefore we had no missings for further analysis. The response rate of the Dutch chiropractors and Belgian chiropractors was 61% and 57% respectively.

## Descriptive statistics

### Attitudes and Beliefs

The biomedical scores of the Dutch respondents were higher on average (51; SD 7) than the scores of the Belgian respondents (45; SD 9). The mean biopsychosocial score on attitudes and beliefs was similar for Dutch (23; SD 3) and Belgian respondents (23; SD 3) (Additional file 5: PABS.PT scores per country).

In Table 2 the overall biomedical and biopsychosocial scores of all the participants are presented, as well as the scores within the three classes identified in LPA (Additional file 3: LPA steps of analysis). These three classes differed primarily on the biomedical scores (Table 2). Therefore, the classes were named 1) high biomedical class (n = 18), 2) mid biomedical class (n = 117) and 3) low biomedical class (n = 23).

**Table 2:** Outcome of orientation in general and per biomedical class following LPA

	Overall (n = 158)	High biomedical class (n = 18)	Mid biomedical class (n = 117)	Low biomedical class (n = 23)
*Biomedical*				
Mean	49	63	49	37
Min – max score	29–76	58—71	41—58	29—41
95% CI		61—65	48—50	36—39
*Biopsychosocial*				
Mean	23	22	23	24
Min – max score	14—35	17—29	16—29	18—29
95% CI		20—23	22—24	23—26

### Characteristics of respondents

The demographic and professional characteristics of the respondents per LPA class are presented in Table 3. The largest group of responders worked in group practice (46.7%). The average age was 42.7 years (SD: 12.7) and 38.3% was female. A high percentage of responders in the low biomedical class worked in Belgium (65.2%), they were slightly older (47.9 years, SD: 16.3) and practiced longer (22.0 years, SD: 15.2) in a solo practice (65.2%) compared to the other two groups.

**Table 3:** Characteristics of responding chiropractors distributed by PABS.PT score

	Total (n = 158)	High biomedical class (n = 18)	Mid biomedical class (n = 117)	Low biomedical class (n = 23)
*Sex (%)*				
Female	38.3	22.2	38.5	39.5
Male	61.7	77.8	61.5	65.2
Age: mean (SD)	42.7 (12.7)	40.6 (8.5)	38.7 (10.9)	47.9 (16.3)
*Country where working (%)*				
The Netherlands	73.3	77.8	80.3	34.8
Belgium	26.7	22.2	19.7	65.2
*Practice type (%)*				
Solo practice	31.1	55.6	39.3	65.2
Group practice	46.7	38.9	47	26.1
Multidisciplinary	17.8	5.6	11.1	8.7
Years in practice: mean (SD)	17.2 (12.5)	13.2 (6.5)	12.5 (9.3)	22.0 (15.2)

### Adherence to guidelines

The overall adherence to guidelines based on the vignettes has been described in two previous articles. Summarising, the majority of the respondents adhered well to the LBP guidelines and to the diagnostic guidelines [26, 27]. Adherence on advice on bedrest was 98,5% in all vignettes [26, 27]. Adherence to guidelines in acute patient vignettes was lower (39.4- 68.2%) than in chronic patient vignettes (62.3–83.8%).

Results from the vignettes suggest that chiropractors in the high biomedical class better adhered to diagnostic imaging guidelines (71.5%) compared to the other two groups (mid biomedical class: 69.1% and low biomedical class: 49.2% respectively) (Tables 4). A similar outcome was observed on the questions in the vignettes on the LBP guidelines on return-to-work, where 81.0% of the high biomedical class adhered to the LBP guidelines, 59.3% of the mid biomedical class and 49.2% of the low biomedical class (Table 5). For activity 93.3% of the high biomedical class adhered to the guidelines, 77.0% of the mid biomedical class, and 64.9% of the low biomedical class (Table 6). There appeared to be no clear differences in adherence to LBP guidelines on low back pain treatment (Table 7).

**Table 4:** Diagnostic imaging guidelines adherence by chiropractors in the vignettes: result of uni-variable logistic mixed model using groups by latent profile analysis based on the PABS.PT score

Overall adherence:	Practice guideline adherence in the vignette (% (95% CI))
For all six vignettes	65.9 (61.5;70.1)
For the three vignettes describing patients with acute low back pain	68.2 (63.4;72.6)
For the three vignettes describing patients with chronic low back pain	62.3 (56.5;67.0)

Uni-variable generalized mixed model (based on all six vignettes)	Adherence on the vignette (% (95% CI))	OR (95% CI)
Latent profile classification:		
High biomedical class	71.5 (57.8;82.0)	2.6 (1.2;5.7)
Mid biomedical class	69.1 (63.8;73.9)	2.3 (1.3;4.0)
Low biomedical class (reference category)	49.2 (36.8;61.7)

**Table 5:** Low back pain guidelines adherence to advice on return to work by chiropractors in the vignettes: result of uni-variable logistic mixed model using groups by latent profile analysis based on the PABS.PT score

Overall adherence:	Low back pain guideline adherence in the vignette (% (95% CI))
For all six vignettes	59.4 (55.2;63.4)
For the three vignettes describing patients with acute low back pain	39.4 (34.6;44.4)
For the three vignettes describing patients with chronic low back pain	81.6. (76.9;85.4)

Uni-variable generalized mixed model (based on all six vignettes) on return to work	Adherence on the vignette (% (95% CI))	OR (95% CI)
*Latent profile classification:*		
High biomedical class	81.0 (70.7;88.3)	4.4 (2.1;9.1)
Mid biomedical class	59.3 (54.5;64.0)	1.5 (0.9;2.4)
Low biomedical class (reference category)	49.2 (38.5;60.0)

**Table 6:** Low back pain guidelines adherence to advice on activity by chiropractors in the vignettes: result of uni-variable logistic mixed model using groups by latent profile analysis based on the PABS.PT score

Overall adherence:	Low back pain guideline adherence in the vignette (% (95% CI))
For all six vignettes	77.5 (72.3;81.9)
For the three vignettes describing patients with acute low back pain	68.1 (61.8;73.8)
For the three vignettes describing patients with chronic low back pain	83.8 (78.8;87.7)

Uni-variable generalized mixed model (based on all six vignettes) on return to work	Adherence on the vignette (% (95% CI))	OR (95% CI)
*Latent profile classification:*		
High biomedical class	93.3 (83.5;97.5)	7.6 (2.2;26.6)
Mid biomedical class	77.0 (70.8;82.3)	1.8 (0.8;4.0)
Low biomedical class (reference category)	64.9 (47.6;79.1)

**Table 7:** Low back pain guidelines adherence to treatment by chiropractors in the vignettes: result of uni-variable logistic mixed model using groups by latent profile analysis based on the PABS.PT score

Overall adherence:	Low back pain guideline adherence in the vignette (% (95% CI))
For all six vignettes	64.5 (58.7;69.9)
For the three vignettes describing patients with acute low back pain	55.9 (50.5;61.1)
For the three vignettes describing patients with chronic low back pain	73.4 (66.7;79.2)

Uni-variable generalized mixed model (based on all six vignettes) on treatment	Adherence on the vignette (% (95% CI))	OR (95% CI)
*Latent profile classification:*		
High biomedical class	48.6 (38.9;73.7)	0.7 (0.3;1.9)
Mid biomedical class	65.9 (58.9;73.9)	1.0 (0.5;2.1)
Low biomedical class (reference category)	66.0 (27.0;79.4)	

The results also suggest that chiropractors in the low biomedical class were more likely to request diagnostic imaging than those in the other two classes (Table 8). Compared to the low biomedical class, diagnostic imaging was less frequently requested by the high biomedical class and the mid biomedical class, respectively 25.3% (95%CI: − 41.7;8.9%) and 8.1%, (95%CI: − 20.0;3.8) less frequent.

**Table 8:** Self-reported frequency of diagnostic imaging. Percentages and results of univariable linear regression analysis for associations between the self-reported amount of requesting of diagnostic imaging and PABS.PT score of chiropractors, using groups by latent profile analysis based on the PABS.PT score

		Total
In what percentage of patients would you like to have diagnostic imaging? (mean %, SD) (n = 158)		42.4 (27.6)

Uni-variable linear regression	Difference (in %) compared to reference group	95% CI
High biomedical class	− 25.3	(− 41.7;− 8.9)
Mid biomedical class	− 8.1	(− 20.0; 3.8)
Low biomedical class (reference category)		

The chiropractors in the low biomedical class appeared to be least familiar with the practice guidelines 52.2%, (95%CI: 35.5;71.2%)), whereas 29.9%, (95%CI: 28.7;38.8%) of the chiropractors in the mid biomedical class and 11.1% (95%CI:2.8;35.5) of the chiropractors in the high biomedical class reported not to be familiar with the practice guidelines (Table 9).

**Table 9:** Self-reported familiarity with practice guidelines in the management of low back pain patients. Percentages and results of univariable logistic regression analysis for associations between familiarity with practice guidelines and reported PABS.PT score of chiropractors, using groups by latent profile analysis based on the PABS.PT score

	Yes	No
Are you familiar with practice guidelines in the management of low back pain patients? (%) (n = 158)	69.0	31.0

Uni-variable logistic regression	% not familiar with practice guidelines (95%CI in %)	OR (95% CI)
High biomedical class (reference category)	11.1 (2.8;35.5)	
Mid biomedical class	29.9 (28.7;38.8)	3.4 (0.8;15.7)
Low biomedical class	52.2 (35.5;71.2)	8.7 (1.6;46.9)

## Discussion

### Summary

This study is the first to investigate the attitudes and beliefs to guideline adherence amongst Dutch and Belgian chiropractors. The majority of responding chiropractors scored midrange on both scales of the PABS.PT, suggesting neither a strong biomedical nor a strong biopsychosocial orientation. In contrast to our hypothesis, the group with a high biomedical score demonstrated better overall adherence to the practice guidelines for the management of LBP and for the guidelines on requesting diagnostic imaging.

### Attitudes and beliefs

The overall observed mean scores on both scales were similar to what was found by Bishop et al. [14]; however, unlike Bishop et al. [14], we found no correlation between the biomedical and biopsychosocial score, which may be the result of our sample. That is, the majority of chiropractic participants scored within the midrange on both scales.

Furthermore, the classes in the LPA were primarily based upon the biomedical orientation of the practitioners as the biopsychosocial orientation did not seem to discriminate between classes. We chose for the three class model based on best fit (AIC and BIC) and as it had better entropy, fitted better to the clinical setting, and gave more information on the high and low biomedical classes than a two class model did.

The average age of respondents in the low biomedical class was slightly higher, with more years of clinical experience, compared to the other two classes. A similar result was found in other studies [28]. It has been suggested that experienced chiropractors may have gained knowledge and experience on the influence of biopsychosocial factors on complaints and feel better equipped to address those factors compared to novice therapists, although this was not reflected in the outcome of the biopsychosocial orientation [28–30]. The difference in age between the groups might be too small, and therefore, not discriminative.

### Adherence

In contrast to our hypothesis, respondents in the high biomedical class, who leaned more towards the biomedical model than the other two groups, showed better adherence to the LBP guidelines and diagnostic imaging guidelines than the low biomedical class. This is also in contrast to what was found in comparable studies, where the group of respondents demonstrating a weaker biomedical orientation showed better adherence to the LBP guidelines and diagnostic imaging guidelines than the stronger biomedically orientated class [14]. Similarly, we found that the low biomedical class reported to request more X-rays in practice than the other two classes.

Reasons why adherence to guideline recommendations was found to be lower in the low biomedical orientated group of responders might be related to several factors, such as the group size, or age. Age in the low biomedical class, as mentioned above, was slightly higher than in the other two classes. In the past, chiropractic educational programs stressed the importance of X-ray use, which is in contrast to current programs which follow the recent diagnostic guidelines. It seems, therefore, likely that older chiropractors are more likely to have had this image-orientated education.

#### Strengths and limitations

The overall response rate was relatively high and comparable to similar chiropractic studies [31, 32]. However, the non-response rate could have led to responder bias, as non-responders may represent a different type of practitioner. It is possible that the attitudes and beliefs, as well as the familiarity with the guidelines are different in this non-responder group compared to the group of responding Belgian and Dutch chiropractors, so caution is urged when interpreting the results. A larger sample size might have identified different (sub)classes/groups. It is possible that the two outliers would have been representative for possible subgroups, that might have stayed undetected due to the current sample size. However, we ran the analyses with and without outliers, which did not lead to different outcomes (Additional file 6: Tables with and without outliers).

A potentially major limitation of this analysis is the year that data were collected, namely in 2013. Although one would expect that the knowledge and application of psychosocial factors has progressed considerably since then, implementation of guidelines in healthcare is a slow process [5, 33–36]. Coenen et al. [36] found that it took 4 out of 5 professionals over 17 years for 80% of the profession to adhere to new guidelines, whereas the 5th group of professionals did not adhere at all [36]. It is not likely that the chiropractic profession differs from other health care professions in this respect. Another potential limitation is the sample size. In general, a minimum number of 500 participants is recommended for LPA [24]. As this number was not met, the distribution of the participants in three groups should be interpreted with caution, which is why further differentiating between the classes, and acute and chronic vignettes was not pursued. Therefore reproducibility of our LPA outcome with larger sample sizes is warranted [24, 37]. Finally, the number of requested X-rays may not reflect the appropriateness of the requested imaging, and could not be examined further because these data were not available.

#### Implications for future research

The chiropractic profession in the Netherlands and Belgium has grown considerably over the years. Currently 390 chiropractors are registered with the Stichting Chiropractie Nederland (SCN) and 166 chiropractors are member of the Belgian Chiropractic Union (BCU). More robust testing of these vignettes on the reliability and validity is advisable for future research. Therefore, a follow-up survey, with the same items among the current group of Dutch and Belgian chiropractors is recommended, to examine the consistency of the results of these vignettes over time. Conducting this survey in other countries will give the opportunity to explore and compare the results amongst chiropractors in other countries. Future research may be used to explore difference in responses between the acute and chronic vignettes. Similarly, future research can investigate the influence of postgraduate education on chiropractors’ attitudes and beliefs as well as on their adherence to guidelines.

Further investigations into the characteristics of Dutch and Belgian chiropractors, compared to other nationalities, may be useful to explore the differences that were found in this study compared to chiropractors in other countries (eg Bishop et al. [14]).

#### Implications for clinical practice

Awareness of guidelines for diagnostic imaging and for LBP guidelines remains important for chiropractors in clinical practice.

## Conclusion

We distinguished three different classes of chiropractors primarily based on their orientation to the biomedical model. In contrast to our hypothesis, the high biomedical group demonstrated better overall adherence to the practice guidelines for the management of LBP and diagnostic imaging. Due to the small size of the classes, the results should be interpreted with caution.

### Supplementary Information

**Additional file 1:** Cross reporting guideline.

**Additional file 2:** Survey

**Additional file 3:** LPA, steps of analysis.

**Additional file 4:** Manual addition of outliers.

**Additional file 5:** PABS.PT scores per country.

**Additional file 6:** Tables with and without outliers.

## Data Availability

The datasets generated and/or analysed during the current study are available from the corresponding author on reasonable request.

## Abbreviations

LBP: Low back pain
PABS.PT: Pain Attitude and Beliefs Scale-Physiotherapists
LPA: Latent Profile Analysis
SMT: Spinal manipulative therapy
SCN: ‘Stichting Chiropractie Nederland’ = Chiropractic Registration Board for the Netherlands
BCU: Belgian Chiropractic Union
OR: Odds Ratio
CI: Confidence Interval
SD: Standard Deviation
