# Supplementary material for: Is the use of diagnostic imaging and the self-reported clinical management of low back pain patients influenced by the attitudes and beliefs of chiropractors? A survey of chiropractors in the Netherlands and Belgium
Source: Chiropr Man Therap. 2024 Jan 8;32:1. doi: 10.1186/s12998-023-00523-y (PMC10775452; doi:10.1186/s12998-023-00523-y)
Supplement: Supplementary file 2 — Additional file 2: Survey [file 12998_2023_523_MOESM2_ESM.pdf]

A survey of the attitudes of Dutch chiropractors towards the use of

Start of Survey

Thank you for participating in this survey.

The survey is about the use of diagnositic imaging by chiropractors in the Netherlands.

The survey will consist of 6 parts.
Part 1: Demographic and practice characteristics
Part 2: Radiographs
Part 3: Opinions
Part 4: Practice Guidelines
Part 5: Clinical Vignettes
Part 6: Attitudes and believes of chiropractors towards back pain

It will take approximately 30-45 minutes to complete the questionnaire and the response will be kept confidential and anonymous.

At ANY time you can pause the questionnaire and return to finish the questionnaire later.

Please press next to start the survey.

Part 1: Demographic and Practice Characteristics

In this section, there are questions about your background and the general characteristics of your practice. The information will be used for data analysis only. Please check the appropriate box, fill in the blank, or circle the appropriate number, as required.

*1. Gender

- ◯ Male
- ◯ Female

*2. Age

Years [ ]

*3. Nationality

- ◯ Dutch
- ◯ Other, please specify:

[ ]

Part 1: Demographic and Practice Characteristics

*4. What year did you graduate from chiropractic college?

Year [ ]

***5. Which chiropractic college did you graduate:**

College:

Country:

***6. When did you start working as a chiropractor?**

Year

***7. Did you have another degree before you started your chiropractic career?**

- No
- Yes, please specify the degree(s):

***8. Have you completed postgraduate chiropractic training?**

- Yes
- No

Part 1: Demographic and Practice Characteristics

***9. Which postgraduate training have you completed?**

- Clinical Sciences
- Radiology
- Sport
- Pediatrics
- Neurology
- Other (please specify)

Part 1: Demographic and Practice Characteristics

***10. Your practice is best described as:**

- Solo practice
- Group practice
- Multidisciplinary setting (with GP, physiotherapist, etc.)
- Other, please specify

Part 1: Demographic and practice characteristics

***11. How many chiropractors are working in your practice?**

Number of chiropractors including yourself

Part 2: Use of diagnostic imaging in chiropractic practice

Part 2 exists of four sections. In the following four sections, there are questions about your use of diagnostic imaging in your practice. In this study diagnostic imaging includes x-rays, MRI scans, CT scans and ultrasound scans.

Please press next to go to the first section

Part 2: Section 1 Requesting existing diagnostic imaging or radiological re...

In the following questions we use the word relevant.

Relevant in this context means:
- the imaging is not older than 5 years
- the imaging gives information about the patients complaint eg. Patient with low back pain has x-rays of his low back and not of a fractured thumb.

***12. Approximately what percentage of your NEW patients have already had relevant diagnostic imaging taken by other health-care professionals (e.g. general practitioner, specialist or colleague chiropractor)?**

Percentage

***13. Do you request the WRITTEN REPORT of the existing (relevant) diagnostic imaging?**

- Never
- Sometimes
- Always

***14. Do you request the EXISTING (relevant) diagnostic imaging?**

- Never
- Sometimes
- Always

Part 2: Section 1 Requesting existing diagnostic imaging or radiological re...

***15. Do you request relevant diagnostic imaging and/or written reports from the radiology department?**

	never	sometimes	always
Yourself	○	○	○
Via the patient	○	○	○
Via the general practitioner	○	○	○

***16. What percentage of diagnostic imaging or written reports, that you request, are: (adds up to 100%)**

% x-rays

% MRI scans

% CT scans

% other e.g. ultrasound scan

Part 2: Section 2 Hospital

In this section, there are questions about the hospitals that send the diagnostic imaging to chiropractors.

***17. How many hospitals are in the referral area of your practice?**

Number of hospitals

***18. From how many of these hospitals, can you get the diagnostic images?**

Number of hospitals

***19. What are the reasons of the hospital for NOT sending you the diagnostic imaging (in some cases)?**

	Yes	No	I don't know
Patient has to request the diagnostic imaging themselves	○	○	○
Chiropractors are not qualified to read diagnostic imaging according to the hospital	○	○	○
It is too expensive to send them	○	○	○

Other (please specify)

20. Do you or the patient have to pay for the requested images?

○ Yes

○ No

Part 2: Section 2 Hospital

***21. How much do you or your patient have to pay the hospital for sending the CD of the diagnostic images to you?**

- Between 0 and 5 Euro
- Between 6 and 10 Euro
- Between 11 and 15 Euro
- Other (please specify)

Part 2: Section 3 Radiographs

In this section, there are questions about how often diagnostic imaging in chiropractic practice is present or is needed.

***22. Of approximately what percentage of your NEW patients would you like to have diagnostic imaging (this includes diagnostic imaging that is already present or referral for diagnostic imaging)?**

Percentage

***23. Do you have one of the following x-ray facilities available at (one of the) practice (s)?**

- X-ray machine
- iDXA
- No

Part 2: Section 3 Radiographs

***24. Of approximately what percentage of your NEW patients of whom you would like to have diagnostic imaging, do you take x-rays scan yourself?**

Example: You would like to have diagnostic imaging in 30% of your new patients. In 50% of these new patients of whom you like to have diagnostic imaging, you take x-rays yourself. Your answer will be 50%.

Percentage

***25. Of approximately what percentage of your NEW patients, of whom you like to have diagnostic imaging, do you refer for diagnostic imaging other than x-rays?**

Example: You would like to have diagnostic imaging in 30% of your new patients. In 5% of these new patients of whom you like to have diagnostic imaging, you refer for diagnostic imaging other than x-rays. Your answer will be 5%.

Percentage

Part 2: Section 3 Radiographs

***26. Of approximately what percentage of your NEW patients of whom you would like to have diagnostic imaging, do you take iDXA scan yourself?**

Example: You would like to have diagnostic imaging in 30% of your new patients. In 50% of these new patients of whom you like to have diagnostic imaging, you take iDXA scan yourself. Your answer will be 50%.

Percentage

***27. Of approximately what percentage of your NEW patients, of whom you like to have diagnostic imaging, do you refer for diagnostic imaging other than iDXA scan?**

Example: You would like to have diagnostic imaging in 30% of your new patients. In 5% of these new patients of whom you like to have diagnostic imaging, you refer for diagnostic imaging other than iDXA scan. Your answer will be 5%.

Percentage

Part 2: Section 3 Radiographs

***28. Of approximately what percentage of your NEW patients of whom you would like to have diagnostic imaging, do you refer for x-rays?**

Example: You would like to have diagnostic imaging in 30% of your new patients. In 50% of these new patients of whom you like to have diagnostic imaging, you refer for x-rays yourself. Your answer will be 50%.

Percentage

***29. Of approximately what percentage of your NEW patients of whom you would like to have diagnostic imaging, do you refer for diagnostic imaging other than x-rays?**

Example: You would like to have diagnostic imaging in 30% of your new patients. In 5% of these new patients of whom you like to have diagnostic imaging, you refer for diagnostic imaging other than x-rays yourself. Your answer will be 5%.

Percentage

Part 2: Section 4 Reasons to use diagnostic imaging in chiropractic practic...

This section contains questions about the use of diagnostic imaging in making clinical decisions.

***30. What are the reasons for using diagnostic imaging and/or a radiological report?**

	Never a reason	Almost never a reason	Sometimes a reason	Often a reason	Always a reason
Diagnosis	○	○	○	○	○
Prognosis	○	○	○	○	○
Exclude contra-indications	○	○	○	○	○
Indicate specific therapy	○	○	○	○	○
Postural Analysis	○	○	○	○	○
Follow-up/ evaluate changes	○	○	○	○	○

Other (please specify)

Part 2: Section 4 Reasons to use diagnostic imaging in chiropractic practic...

***31. What are your considerations to NOT send the patient for diagnostic imaging?**

	Never a reason	Almost never a reason	Sometimes a reason	Often a reason	Always a reason
High radiation load for the patient	○	○	○	○	○
Limited value of imaging information	○	○	○	○	○
No cooperation of the GP	○	○	○	○	○
No cooperation of the hospital	○	○	○	○	○
No cooperation of the patient	○	○	○	○	○
High costs for the patient	○	○	○	○	○
Patient is too young	○	○	○	○	○

Other (please specify)

Part 2: Section 4 Reasons to use diagnostic imaging in chiropractic practic...

***32. What are your considerations to request new diagnostic imaging for patients if recent (< 2 years) diagnostic imaging is present?**

	Never a reason	Almost never a reason	Sometimes a reason	Often a reason	Always a reason
Poor quality of the images	○	○	○	○	○
Images not recent enough in patients with specific conditions eg. scoliosis	○	○	○	○	○
Image not taken in a standing position	○	○	○	○	○
Wrong series or incomplete series of imaging, eg. an Anterior Posterior Open Mouth view is not taken.	○	○	○	○	○
Follow-up after several treatments	○	○	○	○	○
Obvious change in the patients situation or health since the existing images were taken eg. trauma, disease	○	○	○	○	○

Other (please specify)

Part 3 Opinions

You have finished part 2 of the survey.
Part 3 will exist of 2 sections.

Please press next to enter part 3 section 1

Part 3: Section 1 Opinions of chiropractors in the Netherlands on diagnosti...

In this section, there are questions about YOUR opinion on the use of diagnostic imaging in chiropractic practices.

***33. It is important that chiropractors use diagnostic imaging in making clinical decisions about their patients.**

○ agree

○ no opinion

○ disagree

***34. It is important that chiropractors take x-rays themselves in their own practice.**

○ agree

○ no opinion

○ disagree

Part 3: Section 1 Opinions of chiropractors in the Netherlands on diagnosti...

***35. It is important that chiropractors can directly refer for :**

	Agree	No opinion	Disagree
X-rays	◯	◯	◯
MRI scans	◯	◯	◯
CT scan	◯	◯	◯
Other diagnostic imaging eg. Ultrasound	◯	◯	◯

***36. It is important for chiropractors to be able to interpret the following diagnostic imaging themselves:**

	Agree	No opinion	Disagree
X-rays	◯	◯	◯
MRI scans	◯	◯	◯
CT scan	◯	◯	◯
Other diagnostic imaging eg. Ultrasound	◯	◯	◯

Part 3: Section 2 Reading and knowledge of diagnostic imaging by chiropract...

In this section, the questions are about your ability, knowledge and confidence to read diagnostic imaging.

***37. I read .... myself.**

	Never	Sometimes	Always
X-rays	◯	◯	◯
MRI scans	◯	◯	◯
CT scans	◯	◯	◯
Other diagnostic imaging eg. Ultrasound	◯	◯	◯

***38. I feel confident about my ability to read ....**

	Never	Sometimes	Always
X-rays	◯	◯	◯
MRI scans	◯	◯	◯
CT scans	◯	◯	◯
Other diagnostic imaging eg. Ultrasound	◯	◯	◯

Part 3: Section 2 Reading and knowledge of diagnostic imaging by chiropract...

***39. Students who graduate from chiropractic college should be able to take x-rays themselves.**

- Yes
- No

***40. Students who graduate from chiropractic college should be able to interpret x-rays themselves.**

- Yes
- No

***41. Students who graduate from chiropractic college should be able to take MRI scans themselves.**

- Yes
- No

***42. Students who graduate from chiropractic college should be able to interpret MRI scans themselves.**

- Yes
- No

***43. Students who graduate from chiropractic college should be able to take other diagnostic imaging eg. ultrasound scans themselves.**

- Yes
- No

***44. Students who graduate from chiropractic college should be able to interpret other diagnostic imaging eg. ultrasound scans themselves.**

- Yes
- No

***45. Every chiropractor should collect 150 points in 5 years in postgraduate training to keep his registration as a chiropractor by the Stichting Chiropractie Nederland (SCN).**
I believe postgraduate training in radiology (diagnostic imaging) should be mandatory by the Stichting Chiropractie Nederland.

- agree
- neutral
- disagree

Part 3: Section 2 Reading and knowledge of diagnostic imaging by

***46. How many of these 150 points needed every five years, should be mandatory by SCN on postgraduate training in radiology (diagnostic imaging)?**

- 0 points
- 1-5 poinst
- 5-10 points
- 10-15 points
- 15-20 points
- More than 20 points

Part 4: Practice Guidelines

You have finished part 3 of the survey.
Please press next to go to part 4.

Part 4: Practice Guidelines

In this part there are questions about the use of practice guidelines in the management of low back pain patients in your practice.

The following questions are about the GENERAL use of practices quidelines in the management of low back pain patients in your practice.

***47. Are you familiar with any practice guidelines in the management of low back pain patients?**

- Yes
- No

Part 4: Practice Guidelines

***48. Which practice guideline(s) are you familiar with in the management of your low back pain patients**

	Yes	No
AHCPR	○	○
Mercy Guideline	○	○
The Council on Chiropractic Guidelines and Practice Parameters	○	○
British Chiropractic Association Guideline	○	○
CBO	○	○
NHG	○	○
KNGF	○	○
Nice	○	○
Guidelines from the NCA	○	○

Other (please specify)

***49. Do you take the recommendations of these practice guidelines into account in the management of low back pain patients?**

- ○ Yes
- ○ No

Part 4: Practice Guidelines

***50. The recommendations of which practice guidelines do you take into account in the clinical decision making in the management of your low back pain patients? Please tick TWO guidelines you use most often.**

- ☐ AHCPR
- ☐ Mercy Guideline
- ☐ The Council on Chiropractic Guidelines and Practice Parameters
- ☐ British Chiropractic Association Guideline
- ☐ CBO
- ☐ NHG
- ☐ KNGF
- ☐ Nice
- ☐ Guidelines from the NCA
- ☐ Other (please specify)

Part 4: Practice Guidelines

The following questions are on the SPECIFIC use of practice guidelines in the clinical decision making about DIAGNOSTIC IMAGING in your low back patients.

***51. Are you familiar with practice guidelines for sending or requesting diagnostic imaging in low back pain patients?**

- Yes
- No

Part 4: Practice Guidelines

***52. Which practice guideline(s) are you familiar with sending for or requesting diagnostic imaging in your low back patients?**

	Yes	No
Diagnostic imaging guidelines for musculoskeletal complaints in adults		
AHCPR		
Mercy Guideline		
The Council on Chiropractic Guidelines and Practice Parameters		
British Chiropractic Association Guideline		
CBO		
NHG		
KNGF		
Nice		
Guidelines from the NCA		

Other (please specify)

***53. Do you take the recommendations of these practice guidelines into account with sending for or requesting diagnostic imaging in your low back pain patients?**

- Yes
- No

Part 4: Practice Guidelines

***54. The recommendations of which practice guidelines do you take into account with sending for or requesting diagnostic imaging in your low back patients? Please tick TWO guidelines you use most often.**

- [ ] Diagnostic imaging guidelines for musculoskeletal complaints in adults
- [ ] AHCPR
- [ ] Mercy Guideline
- [ ] The Council on Chiropractic Guidelines and Practice Parameters
- [ ] British Chiropractic Association Guideline
- [ ] CBO
- [ ] NHG
- [ ] KNGF
- [ ] Nice
- [ ] Guidelines from the NCA
- [ ] Other (please specify)

Part 5: Clinical Vignettes

You have finished part 4 of the survey. Part 5 will be clinical vignettes.
This section contains three hypothetical scenarios about patients who present to you with acute low back pain and three about patients with chronic low back pain. In the scenarios we have varied a range of features that might influence your management decisions (with regards to investigations you might order and interventions you might recommend or perform). We are aware that the scenario format means that your skills you may normally draw on, such as evaluating non-verbal cues from the patient and performing a physical examination, cannot be a factor in your assessment. Nevertheless, given this understanding, we hope that you address each scenario and answer the questions as best as you can with the information provided.

Please press next to go to the first vignette.

Part 5: Clinical Vignette #1

A 28-year old woman has suffered from acute low back pain for a week. She has been unable to do her job managing a hospital cafeteria for this time. While anxious to return to work, she feels immobilized by the pain. There is no history of trauma. The pain is limited to the low back area, without radiation. On physical examination, there is marked limitation of anterior flexion and tenderness in the left paraspinal region. The neurological examination is normal, with normal straight leg raising to 90 degrees.

The following questions are about what you would GENERALLY do with this patient

***55. Which investigations would you order for this patient at this visit? Please mark all appropriate boxes.**

- [ ] Would not order tests
- [ ] Lumbopelvic X-ray and/or Sacroiliac X-ray series
- [ ] MRI scan
- [ ] CT scan
- [ ] Other advanced Imaging (eg. Ultrasound scan, Bone scan)
- [ ] Lab tests (eg. urinalysis, ESR)
- [ ] Surface Electromyography/nerve conduction
- [ ] Other (please specify)

***56. Which treatments would you offer for this patient at this visit? Please mark all appropriate boxes.**

- [ ] No intervention - expectant observation
- [ ] Chiropractic adjustment (including SMT, Cox, Activator, Gonstead, Thomsen drop)
- [ ] Exercise
- [ ] Education (back school)
- [ ] Massage
- [ ] Spinal traction (not flexion distraction)
- [ ] Psychosocial evaluation by chiropractor
- [ ] Non-exercise modalities (eg. heat, ice etc.)
- [ ] Electrotherapy (eg. TENS, interferential, etc.)
- [ ] Other (please specify)

Part 5: Clinical Vignette #1 continued

A 28-year old woman has suffered from acute low back pain for a week. She has been unable to do her job managing a hospital cafeteria for this time. While anxious to return to work, she feels immobilized by the pain. There is no history of trauma. The pain is limited to the low back area, without radiation. On physical examination, there is marked limitation of anterior flexion and tenderness in the left paraspinal region. The neurological examination is normal, with normal straight leg raising to 90 degrees.

***57. In how many days will be the second appointment?**

Days

*58. Frequency of treatment

How often will you treat this patient?

- ◯ once a week
- ◯ twice a week
- ◯ three times a week
- ◯ Other (please specify)

*59. After how many treatments will you re-evaluate? Re-evaluation means that you monitor the progress not only by asking questions but that you also retest the positive tests found in the physical examination at the first visit.

Treatments

Part 5: Clinical Vignette #1 continued

A 28-year old woman has suffered from acute low back pain for a week. She has been unable to do her job managing a hospital cafeteria for this time. While anxious to return to work, she feels immobilized by the pain. There is no history of trauma. The pain is limited to the low back area, without radiation. On physical examination, there is marked limitation of anterior flexion and tenderness in the left paraspinal region. The neurological examination is normal, with normal straight leg raising to 90 degrees.

*60. What advice would you give the patient at this visit? Please mark all appropriate boxes.

- ☐ No advice
- ☐ Advice on exercise (home programme)
- ☐ General advice on back care
- ☐ Advice to attend a work modification programme
- ☐ Advice to have psychosocial evaluation outside the chiropractors office
- ☐ Other (please specify)
- ☐ Bedrest
- ☐ Lumbar support or corset
- ☐ Suggest over the counter Paracetamol
- ☐ Suggest over the counter NSAIDS

***61. Referral for management outside your office at this visit: Please mark all appropriate boxes.**

- ☐ Would not refer
- ☐ Pain Clinic
- ☐ Refer for prescription Paracetamol
- ☐ Refer for prescrition NSAIDs
- ☐ Refer for prescription Muscle relaxants
- ☐ Refer for prescription Opioids
- ☐ Other Chiropractor
- ☐ Physiotherapy
- ☐ Acupuncture
- ☐ Refer to clinic specializing in formal programs of active supervised exercise & education for back
- ☐ Rehabilitation Clinic
- ☐ General Practitioner
- ☐ Medical Specialist
- ☐ Other (please specify)

Part 5: Clinical Vignette #1 continued

A 28-year old woman has suffered from acute low back pain for a week. She has been unable to do her job managing a hospital cafeteria for this time. While anxious to return to work, she feels immobilized by the pain. There is no history of trauma. The pain is limited to the low back area, without radiation. On physical examination, there is marked limitation of anterior flexion and tenderness in the left paraspinal region. The neurological examination is normal, with normal straight leg raising to 90 degrees.

***62. What kind of medical specialist would you refer?**

- ☐ Neurologist
- ☐ Orthopaedist
- ☐ Rheumatologist
- ☐ Other (please specify)

***63. For what treatment would you refer to a medical specialist?**

- ☐ Injections (eg. trigger point, facet, epidural)
- ☐ Surgery
- ☐ Second Opinion
- ☐ Other (please specify)

*64. Hospitalization

Would you refer the patient for admission to hospital?

- Definitely
- Probably
- Probably not
- Definitely not

Part 5: Clinical Vignette #1 continued

A 28-year old woman has suffered from acute low back pain for a week. She has been unable to do her job managing a hospital cafeteria for this time. While anxious to return to work, she feels immobilized by the pain. There is no history of trauma. The pain is limited to the low back area, without radiation. On physical examination, there is marked limitation of anterior flexion and tenderness in the left paraspinal region. The neurological examination is normal, with normal straight leg raising to 90 degrees.

The following questions are about the SPECIFIC advice you will give this patient.

*65. Return to work

The patient described in the vignette asks what your advice would be about her work. Please tick the one response that best describes what you would recommend this patient to do.
I would recommend this patient to:

- Return to normal work
- Return to part time or light duties
- Be off work until pain has improved
- Be off work until pain has completely disappeared
- Be off work for a further........ weeks (please state number of weeks)

***66. Activity**

The patient described in the vignette asks what your advice would be about activity. Please tick the one response that best describes what you would recommend this patient to do.
I would recommend this patient to:

- Activity Perform usual activities
- Perform activities within the patient’s tolerance
- Perform only pain free activities
- Limit all physical activities until pain disappears

***67. Bedrest**

The patient described in the vignette asks what your advice would be about bedrest. Please tick the one response that best describes what you would recommend this patient to do.
I would recommend this patient to:

- Avoid resting in bed entirely
- Avoid resting in bed as much as possible
- Rest in bed only when pain is severe
- Rest in bed until pain improves substantially
- Rest in bed until pain disappears

Part 5: Clinical vignette #1 end

This is the end of vignette #1.
Please press next to go to clinical vignette #2

Part 5: Clinical Vignette #2

The woman in Vignette # 1 has now had her symptoms for five weeks. You have managed her during this time and there has been little change in pain and no change in physical findings. Today she continues to have pain with movement, and she has not had the confidence to return to work. On examination today, she still has some limitation in the anterior flexion of the spine with a normal neurological examination.

***68. Which investigations would you order for this patient at this visit? Please mark all appropriate boxes.**

- [ ] Would not order tests
- [ ] Lumbopelvic X-ray and/or Sacroiliac X-ray series
- [ ] MRI scan
- [ ] CT scan
- [ ] Other advanced Imaging (eg. Ultrasound scan, Bone scan)
- [ ] Lab tests (eg. urinalysis, ESR)
- [ ] Surface Electromyography/nerve conduction
- [ ] Other (please specify)

***69. Which treatments would you offer for this patient at this visit? Please mark all appropriate boxes.**

- [ ] No intervention - expectant observation
- [ ] Chiropractic adjustment (including SMT, Cox, Activator, Gonstead, Thomsen drop)
- [ ] Exercise
- [ ] Education (back school)
- [ ] Massage
- [ ] Spinal traction (not flexion distraction)
- [ ] Psychosocial evaluation by chiropractor
- [ ] Non-exercise modalities (eg. heat, ice etc.)
- [ ] Electrotherapy (eg. TENS, interferential, etc.)
- [ ] Other (please specify)

Part 5: Clinical Vignette #2 continued

The woman in Vignette # 1 has now had her symptoms for five weeks. You have managed her during this time and there has been little change in pain and no change in physical findings. Today she continues to have pain with movement, and she has not had the confidence to return to work. On examination today, she still has some limitation in the anterior flexion of the spine with a normal neurological examination.

***70. In how many days will be the next appointment?**

Days

***71. Frequency of treatment**

How often will you treat this patient?

- once a week
- twice a week
- three times a week
- Other (please specify)

***72. After how many treatments will you re-evaluate? Re-evaluation means that you monitor the progress not only by asking questions but that you also retest the positive tests found in the physical examination at the first visit.**

Treatments

Part 5: Clinical Vignette #2 continued

The woman in Vignette # 1 has now had her symptoms for five weeks. You have managed her during this time and there has been little change in pain and no change in physical findings. Today she continues to have pain with movement, and she has not had the confidence to return to work. On examination today, she still has some limitation in the anterior flexion of the spine with a normal neurological examination.

***73. What advice would you give the patient at this visit? Please mark all appropriate boxes.**

- No advice
- Advice on exercise (home programme)
- General advice on back care
- Advice to attend a work modification programme
- Advice to have psychosocial evaluation outside the chiropractors office
- Bedrest
- Lumbar support or corset
- Suggest over the counter Paracetamol
- Suggest over the counter NSAIDS
- Other (please specify)

***74. Referral for management outside your office at this visit: Please mark all appropriate boxes.**

- [ ] Would not refer
- [ ] Medical Specialist
- [ ] General Practitioner
- [ ] Pain Clinic
- [ ] Other Chiropractor
- [ ] Physiotherapy
- [ ] Acupuncture
- [ ] Refer to clinic specializing in formal programs of active supervised exercise & education for back
- [ ] Rehabilitation Clinic
- [ ] Refer for prescription Paracetamol
- [ ] Refer for prescrition NSAIDs
- [ ] Refer for prescription Muscle relaxants
- [ ] Refer for prescription Opioids
- [ ] Other (please specify)

Part 5: Clinical Vignette #2 continued

The woman in Vignette # 1 has now had her symptoms for five weeks. You have managed her during this time and there has been little change in pain and no change in physical findings. Today she continues to have pain with movement, and she has not had the confidence to return to work. On examination today, she still has some limitation in the anterior flexion of the spine with a normal neurological examination.

***75. What kind of medical specialist would you refer?**

- [ ] Neurologist
- [ ] Orthopaedist
- [ ] Rheumatologist
- [ ] Other (please specify)

***76. For what treatment would you refer to a medical specialist?**

- [ ] Injections (eg. trigger point, facet, epidural)
- [ ] Surgery
- [ ] Second Opinion
- [ ] Other (please specify)

***77. Hospitalization**

Would you refer the patient for admission to hospital?

- Definitely
- Probably
- Probably not
- Definitely not

Part 5: Clinical Vignette #2 continued

The woman in Vignette # 1 has now had her symptoms for five weeks. You have managed her during this time and there has been little change in pain and no change in physical findings. Today she continues to have pain with movement, and she has not had the confidence to return to work. On examination today, she still has some limitation in the anterior flexion of the spine with a normal neurological examination.

The following questions are about the SPECIFIC advice you will give this patient.

***78. Return to work**

The patient described in the vignette asks what your advice would be about her work. Please tick the one response that best describes what you would recommend this patient to do.
I would recommend this patient to:

- Return to normal work
- Return to part time or light duties
- Be off work until pain has improved
- Be off work until pain has completely disappeared
- Be off work for a further........ weeks (please state number of weeks)

***79. Activity**

The patient described in the vignette asks what your advice would be about activity. Please tick the one response that best describes what you would recommend this patient to do.
I would recommend this patient to:

- Activity Perform usual activities
- Perform activities within the patient’s tolerance
- Perform only pain free activities
- Limit all physical activities until pain disappears

***80. Bedrest**

The patient described in the vignette asks what your advice would be about bedrest. Please tick the one response that best describes what you would recommend this patient to do.
I would recommend this patient to:

- Avoid resting in bed entirely
- Avoid resting in bed as much as possible
- Rest in bed only when pain is severe
- Rest in bed until pain improves substantially
- Rest in bed until pain disappears

Part 5: Clinical Vignette #2 continued

The woman in Vignette # 1 has now had her symptoms for five weeks. You have managed her during this time and there has been little change in pain and no change in physical findings. Today she continues to have pain with movement, and she has not had the confidence to return to work. On examination today, she still has some limitation in the anterior flexion of the spine with a normal neurological examination.

***81. Clinical Vignette # 2 (continued)**

If this patient also reported weight loss and pain at rest five weeks after the first treatment, what (if any) additional investigations, treatments or referral would you order, refer for or recommend?

Part 5: Clinical vignette #2 End

This is the end of vignette #2.
Please press next to go to clinical vignette #3

Part 5: Clinical Vignette #3 continued

A 35-year old auto mechanic presents with a 4 day history of severe acute low back pain with unilateral radiation to the anterior medial leg and the dorsal surface of the foot. The pain started when he was twisting at work but there was no history of trauma. He has some sensory deficit in this distribution and a diminished patella reflex but no motor weakness. Straight leg raising is limited to 45 degrees in the affected leg.

The following questions are about what you would GENERALLY do with this patient

***82. Which investigations would you order for this patient at this visit? Please mark all appropriate boxes.**

- [ ] Would not order tests
- [ ] Lumbopelvic X-ray and/or Sacroiliac X-ray series
- [ ] MRI scan
- [ ] CT scan
- [ ] Other advanced Imaging (eg. Ultrasound scan, Bone scan)
- [ ] Lab tests (eg. urinalysis, ESR)
- [ ] Surface Electromyography/nerve conduction
- [ ] Other (please specify)

***83. Which treatments would you offer for this patient at this visit? Please mark all appropriate boxes.**

- [ ] No intervention - expectant observation
- [ ] Chiropractic adjustment (including SMT, Cox, Activator, Gonstead, Thomsen drop)
- [ ] Exercise
- [ ] Education (back school)
- [ ] Massage
- [ ] Spinal traction (not flexion distraction)
- [ ] Psychosocial evaluation by chiropractor
- [ ] Non-exercise modalities (eg. heat, ice etc.)
- [ ] Electrotherapy (eg. TENS, interferential, etc.)
- [ ] Other (please specify)

Part 5: Clinical Vignette #3 contined

A 35-year old auto mechanic presents with a 4 day history of severe acute low back pain with unilateral radiation to the anterior medial leg and the dorsal surface of the foot. The pain started when he was twisting at work but there was no history of trauma. He has some sensory deficit in this distribution and a diminished patella reflex but no motor weakness. Straight leg raising is limited to 45 degrees in the affected leg.

***84. In how many days will be the second appointment?**

Days

***85. Frequency of treatment**

How often will you treat this patient?

- once a week
- twice a week
- three times a week
- Other (please specify)

***86. After how many treatments will you re-evaluate? Re-evaluation means that you monitor the progress not only by asking questions but that you also retest the positive tests found in the physical examination at the first visit.**

Number of treatments

Part 5: Clinical Vignette #3 continued

A 35-year old auto mechanic presents with a 4 day history of severe acute low back pain with unilateral radiation to the anterior medial leg and the dorsal surface of the foot. The pain started when he was twisting at work but there was no history of trauma. He has some sensory deficit in this distribution and a diminished patella reflex but no motor weakness. Straight leg raising is limited to 45 degrees in the affected leg.

***87. What advice would you give the patient at this visit? Please mark all appropriate boxes.**

- No advice
- Advice on exercise (home programme)
- General advice on back care
- Advice to attend a work modification programme
- Advice to have psychosocial evaluation outside the chiropractors office
- Bedrest
- Lumbar support or corset
- Suggest over the counter Paracetamol
- Suggest over the counter NSAIDS
- Other (please specify)

***88. Referral for management outside your office at this visit: Please mark all appropriate boxes.**

- ☐ Would not refer
- ☐ Medical Specialist
- ☐ General Practitioner
- ☐ Pain Clinic
- ☐ Other Chiropractor
- ☐ Physiotherapy
- ☐ Acupuncture
- ☐ Refer to clinic specializing in formal programs of active supervised exercise & education for back
- ☐ Rehabilitation Clinic
- ☐ Refer for prescription Paracetamol
- ☐ Refer for prescrition NSAIDs
- ☐ Refer for prescription Muscle relaxants
- ☐ Refer for prescription Opioids
- ☐ Other (please specify)

Part 5: Clinical Vignette #3 continued

A 35-year old auto mechanic presents with a 4 day history of severe acute low back pain with unilateral radiation to the anterior medial leg and the dorsal surface of the foot. The pain started when he was twisting at work but there was no history of trauma. He has some sensory deficit in this distribution and a diminished patella reflex but no motor weakness. Straight leg raising is limited to 45 degrees in the affected leg.

***89. What kind of medical specialist would you refer?**

- ☐ Neurologist
- ☐ Orthopaedist
- ☐ Rheumatologist
- ☐ Other (please specify)

***90. For what treatment would you refer to a medical specialist?**

- ☐ Injections (eg. trigger point, facet, epidural)
- ☐ Surgery
- ☐ Second Opinion
- ☐ Other (please specify)

***91. Hospitalization**

Would you refer the patient for admission to hospital?

- Definitely
- Probably
- Probably not
- Definitely not

Part 5: Clinical Vignette #3 continued

A 35-year old auto mechanic presents with a 4 day history of severe acute low back pain with unilateral radiation to the anterior medial leg and the dorsal surface of the foot. The pain started when he was twisting at work but there was no history of trauma. He has some sensory deficit in this distribution and a diminished patella reflex but no motor weakness. Straight leg raising is limited to 45 degrees in the affected leg.

The following questions are about the SPECIFIC advice you will give this patient.

***92. Return to work**

The patient described in the vignette asks what your advice would be about her work. Please tick the one response that best describes what you would recommend this patient to do.
I would recommend this patient to:

- Return to normal work
- Return to part time or light duties
- Be off work until pain has improved
- Be off work until pain has completely disappeared
- Be off work for a further........ weeks (please state number of weeks)

***93. Activity**

The patient described in the vignette asks what your advice would be about activity. Please tick the one response that best describes what you would recommend this patient to do.
I would recommend this patient to:

- Activity Perform usual activities
- Perform activities within the patient's tolerance
- Perform only pain free activities
- Limit all physical activities until pain disappears

***94. Bedrest**

The patient described in the vignette asks what your advice would be about bedrest. Please tick the one response that best describes what you would recommend this patient to do.
I would recommend this patient to:

- Avoid resting in bed entirely
- Avoid resting in bed as much as possible
- Rest in bed only when pain is severe
- Rest in bed until pain improves substantially
- Rest in bed until pain disappears

Part 5: Clinical Vignette #3 continued

A 35-year old auto mechanic presents with a 4 day history of severe acute low back pain with unilateral radiation to the anterior medial leg and the dorsal surface of the foot. The pain started when he was twisting at work but there was no history of trauma. He has some sensory deficit in this distribution and a diminished patella reflex but no motor weakness. Straight leg raising is limited to 45 degrees in the affected leg.

***95. Clinical Vignette # 3 (continued)**

The patient's symptoms and physical examination have NOT improved after you have managed him four weeks. What (if any) additional investigations, treatments or referral would you order, refer for or recommend?

Part 5: Clinical vignette #3 End

This is the end of vignette #3.
Now three chronic patients will be presented to you
Please press next to go to clinical vignette #4.

Part 5: Clinical Vignette #4

A 57 year old office worker sees you for low-back pain. She says her pain began 6 months ago. There was no specific incident that caused the pain. The pain is located in the lower back region, with no radiation. The pain is described as an ache (4 out of 10), with occasional sharp “twinges” with certain movements. The pain is relieved by heat and a massage from her spouse. She has no previous history of low-back pain. The patient is overweight (BMI 30), has mild hypertension, and a family history of type 2 diabetes. The patient rarely does any exercise. On physical examination, there is marked limitation in anterior flexion, extension and rotation and there is tenderness in the paraspinal region on both sides. The neurological examination is normal, with normal straigtht leg raising to 90 degrees. During the consultation she indicates to you that she is anxious that she may have a serious disease. The

patient says “a friend had low-back pain like this and she had an x-ray and it showed that she really had something seriously wrong with her”.

The following questions are about what you would GENERALLY do with this patient

***96. Which investigations would you order for this patient at this visit? Please mark all appropriate boxes.**

- ☐ Would not order tests
- ☐ Lumbopelvic X-ray and/or Sacroiliac X-ray series
- ☐ MRI scan
- ☐ CT scan
- ☐ Other advanced Imaging (eg. Ultrasound scan, Bone scan)
- ☐ Lab tests (eg. urinalysis, ESR)
- ☐ Surface Electromyography/nerve conduction
- ☐ Other (please specify)

***97. Which treatments would you offer for this patient at this visit? Please mark all appropriate boxes.**

- ☐ No intervention - expectant observation
- ☐ Chiropractic adjustment (including SMT, Cox, Activator, Gonstead, Thomsen drop)
- ☐ Exercise
- ☐ Education (back school)
- ☐ Massage
- ☐ Spinal traction (not flexion distraction)
- ☐ Psychosocial evaluation by chiropractor
- ☐ Non-exercise modalities (eg. heat, ice etc.)
- ☐ Electrotherapy (eg. TENS, interferential, etc.)
- ☐ Other (please specify)

Part 5: Clinical Vignette #4 continued

A 57 year old office worker sees you for low-back pain. She says her pain began 6 months ago. There was no specific incident that caused the pain. The pain is located in the lower back region, with no radiation. The pain is described as an ache (4 out of 10), with occasional sharp “twinges” with certain movements. The pain is relieved by heat and a massage from her spouse. She has no history of low-back pain. The patient is overweight (BMI 30), has mild hypertension, and a family history of type 2 diabetes. The patient rarely does any exercise. On physical examination, there is marked limitation in anterior flexion, extension and rotation and there is tenderness in the paraspinal region on both sides. The neurological examination is normal, with normal straigtht leg raising to 90 degrees. During the consultation she indicates to you that she is anxious that she may have a serious disease. The patient says “a friend had low-back pain like this and she had an x-ray and it showed that she really had something seriously wrong with her”. She repeatedly requests an x-ray during the consultation.

***98. In how many days will be the second appointment?**

Days

***99. Frequency of treatment**

How often will you treat this patient?

- once a week
- twice a week
- three times a week
- Other (please specify)

100. After how many treatments will you re-evaluate? Re-evaluation means that you monitor the progress not only by asking questions but that you also retest the positive tests found in the physical examination at the first visit.

Number of treatments

Part 5: Clinical Vignette #4 continued

A 57 year old office worker sees you for low-back pain. She says her pain began 6 months ago. There was no specific incident that caused the pain. The pain is located in the lower back region, with no radiation. The pain is described as an ache (4 out of 10), with occasional sharp “twinges” with certain movements. The pain is relieved by heat and a massage from her spouse. She has no history of low-back pain. The patient is overweight (BMI 30), has mild hypertension, and a family history of type 2 diabetes. The patient rarely does any exercise. On physical examination, there is marked limitation in anterior flexion, extension and rotation and there is tenderness in the paraspinal region on both sides. The neurological examination is normal, with normal straigtht leg raising to 90 degrees. During the consultation she indicates to you that she is anxious that she may have a serious disease. The patient says “a friend had low-back pain like this and she had an x-ray and it showed that she really had something seriously wrong with her”. She repeatedly requests an x-ray during the consultation.

***101. What advice would you give the patient at this visit? Please mark all appropriate boxes.**

- No advice
- Advice on exercise (home programme)
- General advice on back care
- Advice to attend a work modification programme
- Advice to have psychosocial evaluation outside the chiropractors office
- Bedrest
- Lumbar support or corset
- Suggest over the counter Paracetamol
- Suggest over the counter NSAIDS
- Other (please specify)

***102. Referral for management outside your office at this visit: Please mark all appropriate boxes.**

- [ ] Would not refer
- [ ] Medical Specialist
- [ ] General Practitioner
- [ ] Pain Clinic
- [ ] Other Chiropractor
- [ ] Physiotherapy
- [ ] Acupuncture
- [ ] Refer to clinic specializing in formal programs of active supervised exercise & education for back
- [ ] Rehabilitation Clinic
- [ ] Refer for prescription Paracetamol
- [ ] Refer for prescrition NSAIDs
- [ ] Refer for prescription Muscle relaxants
- [ ] Refer for prescription Opioids
- [ ] Other (please specify)

Part 5: Clinical Vignette #4 continued

A 57 year old office worker sees you for low-back pain. She says her pain began 6 months ago. There was no specific incident that caused the pain. The pain is located in the lower back region, with no radiation. The pain is described as an ache (4 out of 10), with occasional sharp "twinges" with certain movements. The pain is relieved by heat and a massage from her spouse. She has no history of low-back pain. The patient is overweight (BMI 30), has mild hypertension, and a family history of type 2 diabetes. The patient rarely does any exercise. On physical examination, there is marked limitation in anterior flexion, extension and rotation and there is tenderness in the paraspinal region on both sides. The neurological examination is normal, with normal straigtht leg raising to 90 degrees. During the consultation she indicates to you that she is anxious that she may have a serious disease. The patient says "a friend had low-back pain like this and she had an x-ray and it showed that she really had something seriously wrong with her". She repeatedly requests an x-ray during the consultation.

***103. What kind of medical specialist would you refer?**

- [ ] Neurologist
- [ ] Orthopaedist
- [ ] Rheumatologist
- [ ] Other (please specify)

***104. For what treatment would you refer to a medical specialist?**

- [ ] Injections (eg. trigger point, facet, epidural)
- [ ] Surgery
- [ ] Second Opinion
- [ ] Other (please specify)

***105. Hospitalization**

Would you refer the patient for admission to hospital?

- Definitely
- Probably
- Probably not
- Definitely not

Part 5: Clinical Vignette #4 continued

A 57 year old office worker sees you for low-back pain. She says her pain began 6 months ago. There was no specific incident that caused the pain. The pain is located in the lower back region, with no radiation. The pain is described as an ache (4 out of 10), with occasional sharp "twinges" with certain movements. The pain is relieved by heat and a massage from her spouse. She has no history of low-back pain. The patient is overweight (BMI 30), has mild hypertension, and a family history of type 2 diabetes. The patient rarely does any exercise. On physical examination, there is marked limitation in anterior flexion, extension and rotation and there is tenderness in the paraspinal region on both sides. The neurological examination is normal, with normal straigtht leg raising to 90 degrees. During the consultation she indicates to you that she is anxious that she may have a serious disease. The patient says "a friend had low-back pain like this and she had an x-ray and it showed that she really had something seriously wrong with her". She repeatedly requests an x-ray during the consultation.

The following questions are about the SPECIFIC advice you will give this patient.

***106. Return to work**

The patient described in the vignette asks what your advice would be about her work. Please tick the one response that best describes what you would recommend this patient to do.
I would recommend this patient to:

- Return to normal work
- Return to part time or light duties
- Be off work until pain has improved
- Be off work until pain has completely disappeared
- Be off work for a further........ weeks (please state number of weeks)

***107. Activity**

The patient described in the vignette asks what your advice would be about activity. Please tick the one response that best describes what you would recommend this patient to do.
I would recommend this patient to:

- Activity Perform usual activities
- Perform activities within the patient's tolerance
- Perform only pain free activities
- Limit all physical activities until pain disappears

***108. Bedrest**

The patient described in the vignette asks what your advice would be about bedrest. Please tick the one response that best describes what you would recommend this patient to do.
I would recommend this patient to:

- Avoid resting in bed entirely
- Avoid resting in bed as much as possible
- Rest in bed only when pain is severe
- Rest in bed until pain improves substantially
- Rest in bed until pain disappears

Part 5: Clinical vignette #4 End

This is the end of vignette #4.
Please press next to go to clinical vignette #5.

Part 5: Clinical Vignette #5

You have managed the woman in Vignette # 4 for five weeks. During this time there has been little change in pain. On examination today, she still has some limitation in the anterior flexion, extension and rotation of the spine with a normal neurological examination.

The following questions are about what you would GENERALLY do with this patient

***109. Which investigations would you order for this patient at this visit? Please mark all appropriate boxes.**

- ☐ Would not order tests
- ☐ Lumbopelvic X-ray and/or Sacroiliac X-ray series
- ☐ MRI scan
- ☐ CT scan
- ☐ Other advanced Imaging (eg. Ultrasound scan, Bone scan)
- ☐ Lab tests (eg. urinalysis, ESR)
- ☐ Surface Electromyography/nerve conduction
- ☐ Other (please specify)

***110. Which treatments would you offer for this patient at this visit? Please mark all appropriate boxes.**

- ☐ No intervention - expectant observation
- ☐ Chiropractic adjustment (including SMT, Cox, Activator, Gonstead, Thomsen drop)
- ☐ Exercise
- ☐ Education (back school)
- ☐ Massage
- ☐ Spinal traction (not flexion distraction)
- ☐ Psychosocial evaluation by chiropractor
- ☐ Non-exercise modalities (eg. heat, ice etc.)
- ☐ Electrotherapy (eg. TENS, interferential, etc.)
- ☐ Other (please specify)

Part 5: Clinical Vignette #5 continued

You have managed the woman in Vignette # 4 for five weeks. During this time there has been little change in pain. On examination today, she still has some limitation in the anterior flexion, extension and rotation of the spine with a normal neurological examination.

***111. In how many days will be the next appointment?**

Days

***112. Frequency of treatment**

How often will you treat this patient?

- once a week
- twice a week
- three times a week
- Other (please specify)

***113. After how many treatments will you re-evaluate? Re-evaluation means that you monitor the progress not only by asking questions but that you also retest the positive tests found in the physical examination at the first visit.**

Number of treatments

Part 5: Clinical Vignette #5 continued

You have managed the woman in Vignette #4 for five weeks. During this time there has been little change in pain. On examination today, she still has some limitation in the anterior flexion, extension and rotation of the spine with a normal neurological examination.

***114. What advice would you give the patient at this visit? Please mark all appropriate boxes.**

- No advice
- Advice on exercise (home programme)
- General advice on back care
- Advice to attend a work modification programme
- Advice to have psychosocial evaluation outside the chiropractors office
- Bedrest
- Lumbar support or corset
- Suggest over the counter Paracetamol
- Suggest over the counter NSAIDS
- Other (please specify)

***115. Referral for management outside your office at this visit: Please mark all appropriate boxes.**

- ☐ Would not refer
- ☐ Medical Specialist
- ☐ General Practitioner
- ☐ Pain Clinic
- ☐ Other Chiropractor
- ☐ Physiotherapy
- ☐ Acupuncture
- ☐ Refer to clinic specializing in formal programs of active supervised exercise & education for back
- ☐ Rehabilitation Clinic
- ☐ Refer for prescription Paracetamol
- ☐ Refer for prescrition NSAIDs
- ☐ Refer for prescription Muscle relaxants
- ☐ Refer for prescription Opioids
- ☐ Other (please specify)

Part 5: Clinical Vignette #5 continued

You have managed the woman in Vignette # 4 for five weeks. During this time there has been little change in pain. On examination today, she still has some limitation in the anterior flexion, extension and rotation of the spine with a normal neurological examination.

***116. What kind of medical specialist would you refer?**

- ☐ Neurologist
- ☐ Orthopaedist
- ☐ Rheumatologist
- ☐ Other (please specify)

***117. For what treatment would you refer to a medical specialist?**

- ☐ Injections (eg. trigger point, facet, epidural)
- ☐ Surgery
- ☐ Second Opinion
- ☐ Other (please specify)

118. Hospitalization

Would you refer the patient for admission to hospital?

- Definitely
- Probably
- Probably not
- Definitely not

Part 5: Clinical Vignette #5 continued

You have managed the woman in Vignette # 4 for five weeks. During this time there has been little change in pain. On examination today, she still has some limitation in the anterior flexion, extension and rotation of the spine with a normal neurological examination.

The following questions are about the SPECIFIC advice you will give this patient.

***119. Return to work**

The patient described in the vignette asks what your advice would be about her work. Please tick the one response that best describes what you would recommend this patient to do).

- Return to normal work
- Return to part time or light duties
- Be off work until pain has improved
- Be off work until pain has completely disappeared
- Be off work for a further........ weeks (please state number of weeks)

***120. Activity**

The patient described in the vignette asks what your advice would be about activity. Please tick the one response that best describes what you would recommend this patient to do.
I would recommend this patient to:

- Activity Perform usual activities
- Perform activities within the patient’s tolerance
- Perform only pain free activities
- Limit all physical activities until pain disappears

***121. Bedrest**

The patient described in the vignette asks what your advice would be about bedrest. Please tick the one response that best describes what you would recommend this patient to do.
I would recommend this patient to:

- ○ Avoid resting in bed entirely
- ○ Avoid resting in bed as much as possible
- ○ Rest in bed only when pain is severe
- ○ Rest in bed until pain improves substantially
- ○ Rest in bed until pain disappears

Part 5: Clinical vignette #5 End

This is the end of vignette #5.
Please press next to go to clinical vignette #6.

Part 5: Clinical Vignette #6

A 50-year old real estate agent consults you for his low-back pain with radiation to the left leg especially the posterior calf and lateral foot. The pain has been present for 12 weeks, starting two days after a weekend of heavy work in the garden. Today he describes the pain as a sharp pain (5 to 6 out of 10). He has had previous, similar episodes of low-back pain with radiation to the leg that have lasted one to two weeks. He has no other health concerns. On physical examination, he has some sensory deficit in the posterior calf and lateral foot and a diminished ankle reflex but no motor weakness. Straight leg raising is limited to 45 degrees in the affected leg.

The following questions are about what you would GENERALLY do with this patient

***122. Which investigations would you order for this patient at this visit? Please mark all appropriate boxes.**

- ☐ Would not order tests
- ☐ Lumbopelvic X-ray and/or Sacroiliac X-ray series
- ☐ MRI scan
- ☐ CT scan
- ☐ Other advanced Imaging (eg. Ultrasound scan, Bone scan)
- ☐ Lab tests (eg. urinalysis, ESR)
- ☐ Surface Electromyography/nerve conduction
- ☐ Other (please specify)

***123. Which treatments would you offer for this patient at this visit? Please mark all appropriate boxes.**

- [ ] No intervention - expectant observation
- [ ] Chiropractic adjustment (including SMT, Cox, Activator, Gonstead, Thomsen drop)
- [ ] Exercise
- [ ] Education (back school)
- [ ] Massage
- [ ] Spinal traction (not flexion distraction)
- [ ] Psychosocial evaluation by chiropractor
- [ ] Non-exercise modalities (eg. heat, ice etc.)
- [ ] Electrotherapy (eg. TENS, interferential, etc.)
- [ ] Other (please specify)

Part 5: Clinical Vignette #6 continued

A 50-year old real estate agent consults you for his low-back pain with radiation to the left leg especially the posterior calf and lateral foot. The pain has been present for 12 weeks, starting two days after a weekend of heavy work in the garden. Today he describes the pain as a sharp pain (5 to 6 out of 10). He has had previous, similar episodes of low-back pain with radiation to the leg that have lasted one to two weeks. He has no other health concerns. On physical examination, he has some sensory deficit in the posterior calf and lateral foot and a diminished ankle reflex but no motor weakness. Straight leg raising is limited to 45 degrees in the affected leg.

***124. In how many days will be the second appointment?**

Days

***125. Frequency of treatment**

How often will you treat this patient?

- once a week
- twice a week
- three times a week
- Other (please specify)

***126. After how many treatments will you re-evaluate?Re-evaluation means that you monitor the progress not only by asking questions but that you also retest the positive tests found in the physical examination at the first visit.**

Number of treatments

Part 5: Clinical Vignette #6 continued

A 50-year old real estate agent consults you for his low-back pain with radiation to the left leg especially the posterior

calf and lateral foot. The pain has been present for 12 weeks, starting two days after a weekend of heavy work in the garden. Today he describes the pain as a sharp pain (5 to 6 out of 10). He has had previous, similar episodes of low-back pain with radiation to the leg that have lasted one to two weeks. He has no other health concerns. On physical examination, he has some sensory deficit in the posterior calf and lateral foot and a diminished ankle reflex but no motor weakness. Straight leg raising is limited to 45 degrees in the affected leg.

***127. What advice would you give the patient at this visit? Please mark all appropriate boxes.**

- ☐ No advice
- ☐ Advice on exercise (home programme)
- ☐ General advice on back care
- ☐ Advice to attend a work modification programme
- ☐ Advice to have psychosocial evaluation outside the chiropractors office
- ☐ Bedrest
- ☐ Lumbar support or corset
- ☐ Suggest over the counter Paracetamol
- ☐ Suggest over the counter NSAIDS
- ☐ Other (please specify)

***128. Referral for management outside your office at this visit: Please mark all appropriate boxes.**

- ☐ Would not refer
- ☐ Medical Specialist
- ☐ General Practitioner
- ☐ Other Chiropractor
- ☐ Physiotherapy
- ☐ Refer to clinic specializing in formal programs of active supervised exercise & education for back
- ☐ Pain Clinic
- ☐ Acupuncture
- ☐ Rehabilitation Clinic
- ☐ Refer for prescription Paracetamol
- ☐ Refer for prescrition NSAIDs
- ☐ Refer for prescription Muscle relaxants
- ☐ Refer for prescription Opioids
- ☐ Other (please specify)

Part 5: Clinical Vignette #6 continued

A 50-year old real estate agent consults you for his low-back pain with radiation to the left leg especially the posterior calf and lateral foot. The pain has been present for 12 weeks, starting two days after a weekend of heavy work in the garden. Today he describes the pain as a sharp pain (5 to 6 out of 10). He has had previous, similar episodes of low-back pain with radiation to the leg that have lasted one to two weeks. He has no other health concerns. On physical examination, he has some sensory deficit in the posterior calf and lateral foot and a diminished ankle reflex but no motor weakness. Straight leg raising is limited to 45 degrees in the affected leg.

*129. What kind of medical specialist would you refer?

- [ ] Neurologist
- [ ] Orthopaedist
- [ ] Rheumatologist
- [ ] Other (please specify)

*130. For what treatment would you refer to a medical specialist?

- [ ] Injections (eg. trigger point, facet, epidural)
- [ ] Surgery
- [ ] Second Opinion
- [ ] Other (please specify)

*131. Hospitalization

Would you refer the patient for admission to hospital?

- ( ) Definitely
- ( ) Probably
- ( ) Probably not
- ( ) Definitely not

Part 5: Clinical Vignette #6 continued

A 50-year old real estate agent consults you for his low-back pain with radiation to the left leg especially the posterior calf and lateral foot. The pain has been present for 12 weeks, starting two days after a weekend of heavy work in the garden. Today he describes the pain as a sharp pain (5 to 6 out of 10). He has had previous, similar episodes of low-back pain with radiation to the leg that have lasted one to two weeks. He has no other health concerns. On physical examination, he has some sensory deficit in the posterior calf and lateral foot and a diminished ankle reflex but no motor weakness. Straight leg raising is limited to 45 degrees in the affected leg.

The following questions are about the SPECIFIC advice you will give this patient.

***132. Return to work**

The patient described in the vignette asks what your advice would be about her work. Please tick the one response that best describes what you would recommend this patient to do.
I would recommend this patient to:

- Return to normal work
- Return to part time or light duties
- Be off work until pain has improved
- Be off work until pain has completely disappeared
- Be off work for a further........ weeks (please state number of weeks)

***133. Activity**

The patient described in the vignette asks what your advice would be about activity. Please tick the one response that best describes what you would recommend this patient to do.
I would recommend this patient to:

- Activity Perform usual activities
- Perform activities within the patient’s tolerance
- Perform only pain free activities
- Limit all physical activities until pain disappears

***134. Bedrest**

The patient described in the vignette asks what your advice would be about bedrest. Please tick the one response that best describes what you would recommend this patient to do.
I would recommend this patient to:

- Avoid resting in bed entirely
- Avoid resting in bed as much as possible
- Rest in bed only when pain is severe
- Rest in bed until pain improves substantially
- Rest in bed until pain disappears

Part 5: Clinical Vignette #6 continued

A 50-year old real estate agent consults you for his low-back pain with radiation to the left leg especially the posterior calf and lateral foot. The pain has been present for 12 weeks, starting two days after a weekend of heavy work in the garden. Today he describes the pain as a sharp pain (5 to 6 out of 10). He has had previous, similar episodes of low-back pain with radiation to the leg that have lasted one to two weeks. He has no other health concerns. On physical examination, he has some sensory deficit in the posterior calf and lateral foot and a diminished ankle reflex but no motor weakness. Straight leg raising is limited to 45 degrees in the affected leg.

***135. Clinical Vignette # 6 (continued)**

The patient described in Vignette #6 now comes in on a very busy day at the practice and there are many patients already in the waiting room wanting to see you. The patient has seen you weekly over the last four weeks for her low-back pain and complains that it is not improving. He is frustrated with his lack of improvement and thinks something different needs to be done. He is dissatisfied that he has not already been referred for further investigation, and insists that you refer him now.

What (if any) additional investigations, treatments or referral would you order, refer for or recommend?

Part 5: Clinical vignette #6 End

This is the end of vignette #6.
Please press next to go to Part 6 (the last part).

Part 6: Attitudes and beliefs of chiropractic towards back pain

This section contains questions about your perception of back pain.

*136. Perception of back pain

	Totally agree	Largely agree	Agree to some extend	Disagree to some extend	Largely disagree	Totally disagree
Back pain sufferers should refrain from all physical activity in order to avoid injury						
Good posture prevents back pain						
Knowledge of the tissue damage is not necessary for effective therapy						
Reduction of daily physical exertion is a significant factor in treating back pain						
Not enough effort is made to find the underlying organic causes of back pain						
Mental stress can cause back pain even in the absence of tissue damage						
The cause of back pain is unknown						
Unilateral physical stress is not a cause of back pain						
Patients who have suffered back pain should avoid activities that stress the back						
Pain is a nociceptive stimulus, indicating tissue damage						

Part 6: Attitudes and beliefs of chiropractic towards back pain

*137. Perception of back pain

	Totally agree	Largely agree	Agree to some extend	Disagree to some extend	Largely disagree	Totally disagree
A patient suffering from severe back pain will benefit from physical exercise						
Functional limitations associated with back pain are the result of psychosocial factors						
The best advice for back pain is: ‘Take care’ and ‘Make no unnecessary movements’						
Patients with back pain should preferably practice only pain free movements						
Back pain indicates that there is something dangerously wrong with the back						
The way patients view their pain influences the progress of the symptoms						
Therapy may have been successful even if pain remains						
Therapy can completely alleviate the functional symptoms caused by back pain						
If ADL activities cause more back pain, this is not dangerous						
Back pain indicates the presence of organic injury						

Part 6: Attitudes and beliefs of chiropractic towards back pain

*138. Perception of back pain

	Totally agree	Largely agree	Agree to some extend	Disagree to some extend	Largely disagree	Totally disagree
Sport should not be recommended for patients with back pain	○	○	○	○	○	○
If back pain increases in severity, I immediately adjust the intensity of my treatment accordingly	○	○	○	○	○	○
If therapy does not result in a reduction in back pain, there is a high risk of severe restrictions in the long term	○	○	○	○	○	○
Pain reduction is a precondition for the restoration of normal functioning	○	○	○	○	○	○
Increased pain indicates new tissue damage or the spread of existing damage	○	○	○	○	○	○
It is the task of the chiropractor to remove the cause of back pain	○	○	○	○	○	○
There is no effective treatment to eliminate back pain	○	○	○	○	○	○
TENS and/or back braces support functional recovery	○	○	○	○	○	○

Part 6: Attitudes and beliefs of chiropractic towards back pain

*139. Perception of back pain

	Totally agree	Largely agree	Agree to some extend	Disagree to some extend	Largely disagree	Totally disagree
Even if the pain has worsened, the intensity of the next treatment can be increased	○	○	○	○	○	○
If patients complain of pain during exercise, I worry that damage is being caused	○	○	○	○	○	○
The severity of tissue damage determines the level of pain	○	○	○	○	○	○
A rapid resumption of daily activities is an important goal of the treatment	○	○	○	○	○	○
Learning to cope with stress promotes recovery from back pain	○	○	○	○	○	○
Exercises that may be back straining should not be avoided during the treatment	○	○	○	○	○	○
In the long run, patients with back pain have a higher risk of developing spinal impairments	○	○	○	○	○	○
In back pain, imaging tests are unnecessary	○	○	○	○	○	○

End of Survey

Thank you very much for taking the time to fill out this questionnaire.
