# Supplementary material for: Is the use of diagnostic imaging and the self-reported clinical management of low back pain patients influenced by the attitudes and beliefs of chiropractors? A survey of chiropractors in the Netherlands and Belgium
Source: Chiropr Man Therap. 2024 Jan 8;32:1. doi: 10.1186/s12998-023-00523-y (PMC10775452; doi:10.1186/s12998-023-00523-y)
Supplement: Supplementary file 3 — Additional file 3: LPA, steps of analysis. [file 12998_2023_523_MOESM3_ESM.docx]

**Appendix 2 LPA steps of analysis:**

LPA steps of analysis:

Mode of conduct

The following steps were taken:

- First the distribution of all outcomes was observed
- The models were run first with all participants, then the models were run without the outliers (n=2).
  - The models without the outliers gave a different outcome; the outcome was disproportionately influenced by the two outliers, therefore the outliers were taken out to determine the classes.
- Four different models were run: a) restricted/basic, b) with several variations/variances, c) with several covariances of residues and d) a combination of b) and c).

4 different models were run: a)restricted/basis, b) different variances c) with co-variances of residuals d) combination of b+ c

- Several models were run with random starts to see if this made any difference

Based on AIC and BIC the best models were chosen

- Outcomes for 4 class and 5 class were rejected as the outcome would be unreliable and unstable.

2 class (restricted) outcome

|  | Class 1 (N=93) | Class 2 (N=63) |
| --- | --- | --- |
| Biomedical score (average/mean) | 48.3 | 50.0 |
| Biopsychosocial score (mean) | 24.9 | 20.7 |

3 class model (restricted) outcome

|  | Class 1 (N=17) | Class 2 (N=116) | Class 3 (N=23) |
| --- | --- | --- | --- |
| Biomedical score (average) | 61.9 | 49.1 | 38.8 |
| Biopsychosocial score (average) | 22.1 | 23.2 | 24.0 |

Fit indices 2 and 3 class model and entropy* of models

|  | AIC |  | BIC | entropy |
| --- | --- | --- | --- | --- |
| 2 class model | 1868.0 |  | 1889.4 | 0.47 |
| 3 class model | 1873.5 |  | 1904.0 | 0.73 |

*entropy preferably > 0.8, but interpretation is dependent on context

Followed by:

- Model was chosen
- Another round of checking with more random starts
- Seed used for reproducability
