# Supplementary material for: Is the use of diagnostic imaging and the self-reported clinical management of low back pain patients influenced by the attitudes and beliefs of chiropractors? A survey of chiropractors in the Netherlands and Belgium
Source: Chiropr Man Therap. 2024 Jan 8;32:1. doi: 10.1186/s12998-023-00523-y (PMC10775452; doi:10.1186/s12998-023-00523-y)
Supplement: Supplementary file 4 — Additional file 4: Manual addition of outliers. [file 12998_2023_523_MOESM4_ESM.docx]

**Appendix 3 manual addition of outliers:**

The two outliers were added to the 3 class model. The first outlier fitted well in the high biomedical class (score of 76 biomedical and 14 biopsychosocial on the PABS.PT score). The second outlier (score of 44 biomedical and 35 biopsychosocial on the PABS.PT score) was placed in the mid biomedical class, based mainly on its biomedical score.
