# Supplementary material for: Is the use of diagnostic imaging and the self-reported clinical management of low back pain patients influenced by the attitudes and beliefs of chiropractors? A survey of chiropractors in the Netherlands and Belgium
Source: Chiropr Man Therap. 2024 Jan 8;32:1. doi: 10.1186/s12998-023-00523-y (PMC10775452; doi:10.1186/s12998-023-00523-y)
Supplement: Supplementary file 5 — Additional file 5: PABS.PT scores per country. [file 12998_2023_523_MOESM5_ESM.docx]

**Appendix 4 PABS.PT scores per country**

| Country where PABS.PT was taken | |  | Minimum | Maximum | Mean | Standard deviation |
| --- | --- | --- | --- | --- | --- | --- |
| The Netherlands | Biomedical score | | 35,00 | 76,00 | 50,5431 | 7,13155 |
| N = 116 | Biopsychosocial score | | 14,00 | 35,00 | 23,1293 | 3,14996 |
| Belgium | Biomedical score | | 29,00 | 68,00 | 45,3095 | 8,76659 |
| N = 42 | Biopsychosocial score | | 16,00 | 29,00 | 23,3810 | 3,29784 |
