# Supplementary material for: Is the use of diagnostic imaging and the self-reported clinical management of low back pain patients influenced by the attitudes and beliefs of chiropractors? A survey of chiropractors in the Netherlands and Belgium
Source: Chiropr Man Therap. 2024 Jan 8;32:1. doi: 10.1186/s12998-023-00523-y (PMC10775452; doi:10.1186/s12998-023-00523-y)
Supplement: Supplementary file 6 — Additional file 6: Tables with and without outliers. [file 12998_2023_523_MOESM6_ESM.docx]

**Appendix 6 Tables with OR (95% CI) with and without outliers**

| Table 4a. Diagnostic imaging guidelines adherence by chiropractors in the vignettes: result of uni-variable logistic mixed model using groups by latent profile analysis based on the PABS.PT score. | | |
| --- | --- | --- |
| Uni-variable generalized mixed model (based on all six vignettes) with outliers |  | **OR (95% CI)** |
| Latent profile classification: |  |  |
| High biomedical class |  | 2.6 (1.2;5.7) |
| Mid biomedical class |  | 2.3 (1.3;4.0) |
| Low biomedical class (reference category) |  |  |
|  |  |  |
| Uni-variable generalized mixed model (based on all six vignettes) without outliers |  | **OR (95% CI)** |
| Latent profile classification: |  |  |
| High biomedical class |  | 2.5 (1.1;5.6) |
| Mid biomedical class |  | 2.3 (1.3;4.0) |
| Low biomedical class (reference category) |  |  |

| Table 4b. Low back pain guidelines adherence to treatment by chiropractors in the vignettes: result of uni-variable logistic mixed model using groups by latent profile analysis based on the PABS.PT score. | | |  |
| --- | --- | --- | --- |
| Uni-variable generalized mixed model (based on all six vignettes) on treatment with outliers |  | **OR (95% CI)** |  |
| Latent profile classification: |  |  |  |
| High biomedical class |  | 0.7 (0.3;1.9) |  |
| Mid biomedical class |  | 1.0 (0.5;2.1) |  |
| Low biomedical class (reference category) |  |  |  |
|  |  |  |  |
| Uni-variable generalized mixed model (based on all six vignettes) on treatment without outliers |  | **OR (95% CI)** |  |
| Latent profile classification: |  |  |  |
| High biomedical class |  | 0.8 (0.3;2.1) |  |
| Mid biomedical class |  | 1.0 (0.5;2.1) |  |
| Low biomedical class (reference category) |  |  |  |
|  |  |  |  |
| Table 4c. Low back pain guidelines adherence to advice on return to work by chiropractors in the vignettes: result of uni-variable logistic mixed model using groups by latent profile analysis based on the PABS.PT score. | | |  |
| Uni-variable generalized mixed model (based on all six vignettes) on return to work with outliers |  | **OR (95% CI)** |  |
| Latent profile classification: |  |  |  |
| High biomedical class |  | 4.4 (2.1;9.1) |  |
| Mid biomedical class |  | 1.5 (0.9;2.4) |  |
| Low biomedical class (reference category) |  |  |  |
|  |  |  |  |
| Uni-variable generalized mixed model (based on all six vignettes) on return to work without outliers | |  | **OR (95% CI)** |
| Latent profile classification: | |  |  |
| High biomedical class | |  | 4.4 (2.1;9.0) |
| Mid biomedical class | |  | 1.5 (1.0;2.5) |
| Low biomedical class (reference category) | |  |  |

| Table 4d Low back pain guidelines adherence to advice on activity by chiropractors in the vignettes: result of uni-variable logistic mixed model using groups by latent profile analysis based on the PABS.PT score. | | |
| --- | --- | --- |
| Uni-variable generalized mixed model (based on all six vignettes) on activity with outliers |  | **OR (95% CI)** |
| Latent profile classification: |  |  |
| High biomedical class |  | 7.6 (2.2;26.6) |
| Mid biomedical class |  | 1.8 (0.8;4.0) |
| Low biomedical class (reference category) |  |  |

| Uni-variable generalized mixed model (based on all six vignettes) on activity without outliers |  | OR (95% CI) |
| --- | --- | --- |
| Latent profile classification: |  |  |
| High biomedical class |  | 7.1 (2.0;24.4) |
| Mid biomedical class |  | 1.9 (0.9;4.1) |
| Low biomedical class (reference category) |  |  |

**Table 5a. Self-reported frequency of diagnostic imaging. Percentages and results of univariable linear regression analysis for associations between the self-reported amount of requesting of diagnostic imaging and PABS.PT score of chiropractors, using groups by latent profile analysis based on the PABS.PT score.**

| **Uni-variable linear regression with outliers** | Difference (in %) compared to reference group | 95% CI |
| --- | --- | --- |
| **High biomedical class** | -25.3 | (-41.7;-8.9) |
| **Mid biomedical class** | -8.1 | (-20.0; 3.8) |
| **Low biomedical class (reference category)** |  |  |

| **Uni-variable linear regression without outliers** | Difference (in %) compared to reference group | 95% CI |
| --- | --- | --- |
| **High biomedical class** | -24.3 | (-40.9;-7.7) |
| **Mid biomedical class** | -7.7 | (-19.5; 4.1) |
| **Low biomedical class (reference category)** |  |  |

**Table 5b. Self-reported familiarity with practice guidelines in the management of low back pain patients. Percentages and results of univariable logistic regression analysis for associations between familiarity with practice guidelines and reported PABS.PT score of chiropractors, using groups by latent profile analysis based on the PABS.PT score.**

| **Uni-variable logistic regression with outliers** |  | **OR (95% CI)** |
| --- | --- | --- |
| **High biomedical class (reference category)** |  |  |
| **Mid biomedical class** |  | 3.4 (0.8;15.7) |
| **Low biomedical class** |  | 8.7 (1.6;46.9) |

| **Uni-variable logistic regression without outliers** |  | **OR (95% CI)** |
| --- | --- | --- |
| **High biomedical class (reference category)** |  |  |
| **Mid biomedical class** |  | 3.2 (0.7;14.9) |
| **Low biomedical class** |  | 8.7 (1.5;44.2) |
